# Exploratory Food-Frequency Scores and Self-Reported Asthma in Italian Adults: A Nationwide Cross-Sectional Study

**DOI:** 10.3390/nu18213485

**Published:** 2026-10-23

**Authors:** Manuela Chiavarini, Jacopo Dolcini, Giorgio Firmani, Pamela Barbadoro

**Affiliations:** 1 Department of Health Sciences, University of Florence, Viale G.B. Morgagni 48, 50134 Florence, Italy; 2 Department of Biomedical Sciences and Public Health, Section of Hygiene, Preventive Medicine and Public Health, Polytechnic University of the Marche Region, Via Tronto 10/A, 60126 Ancona, Italy

**Keywords:** self-reported asthma, food-frequency scores, dietary patterns, nutrition, epidemiology, Italy, cross-sectional study

## Abstract

Background: Associations between diet and asthma in adults remain uncertain. Objective: To examine associations between two exploratory food-frequency scores and self-reported asthma. Methods: We analysed public-use data from the 2023 Italian Aspects of Daily Life survey. The PRO and ANTI scores summarised selected food frequencies; neither was a validated measure of dietary inflammatory potential. Survey-weighted logistic models were adjusted for age group, sex, education, body mass index, smoking, and household economic resources. Continuous and tertile specifications were fitted separately; an additional model included both continuous scores. Results: Among 33,182 adults with asthma information, weighted prevalence was 4.62% (95% CI 4.37–4.89). The regression sample included 32,000 adults and 1460 asthma cases. In the mutually adjusted model, PRO was not associated with asthma (OR per 10 points 1.004, 95% CI 0.952–1.059), whereas ANTI was inversely associated (OR 0.947, 95% CI 0.907–0.989). ANTI tertile comparisons did not reach conventional statistical significance. Sex-specific and restricted-sample analyses were exploratory. Conclusions: A modest inverse association was observed for the continuous ANTI score, but evidence was sensitive to exposure parameterisation. These cross-sectional findings do not demonstrate reduced asthma risk, respiratory benefit, or an inflammatory mechanism. Replication with validated dietary assessment and clinically characterised asthma is needed.

## 1. Introduction

Asthma is a major public health challenge. A Global Burden of Disease 2021 analysis reported a global prevalence of 3340 cases per 100,000 people [1]. Genetic susceptibility and environmental exposures contribute to its heterogeneous pathophysiology [2]. Diet is one potentially relevant exposure, but observational diet–asthma associations do not by themselves establish a causal or inflammatory mechanism.

**Figure 1:**
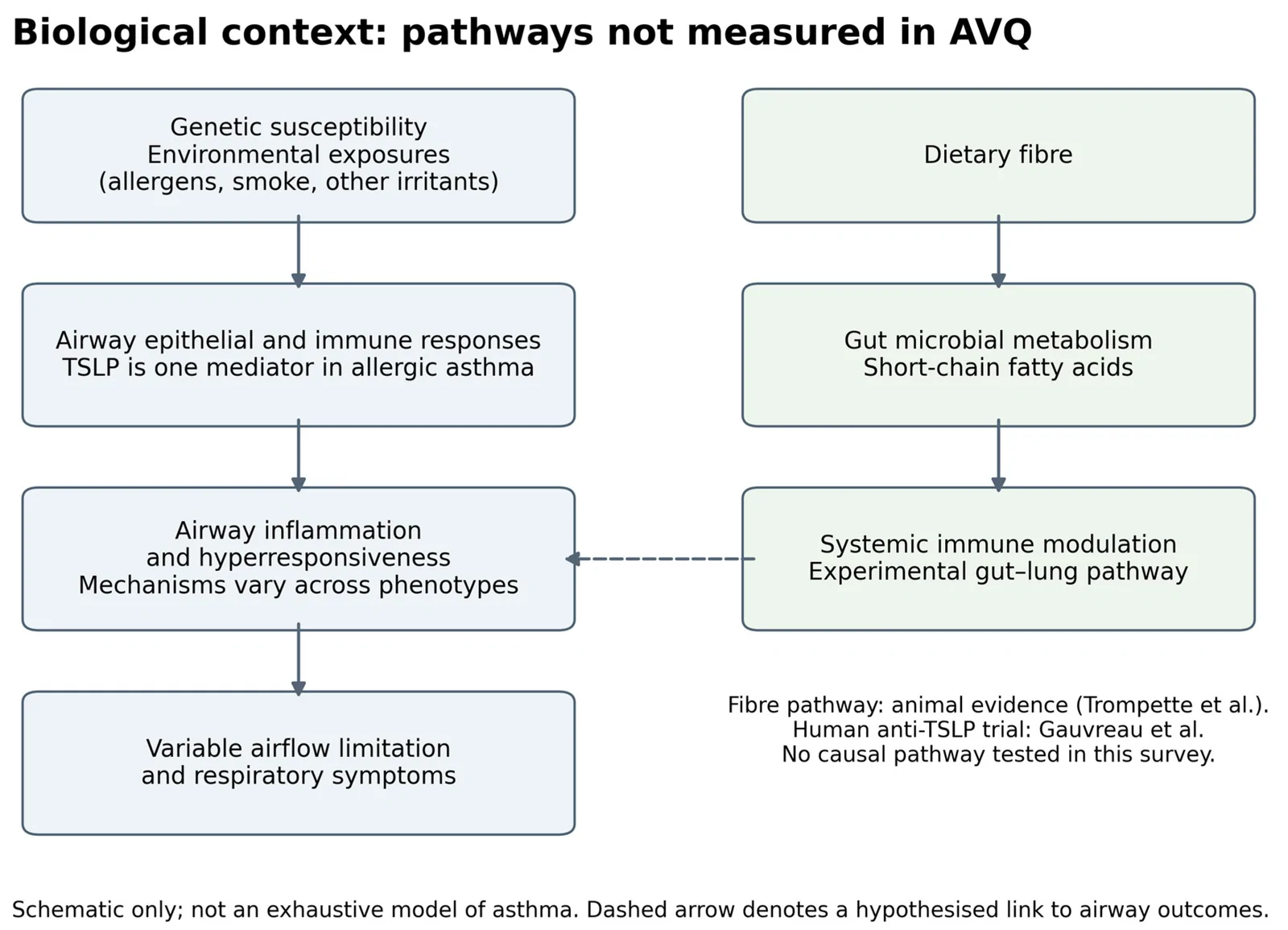
Simplified biological context for asthma and a possible diet–microbiota pathway. The schematic summarises background evidence [2,3,4], not findings of the AVQ analysis. The microbial pathway shown is supported by experimental animal work and cannot be extrapolated directly to these food-frequency scores. TSLP: thymic stromal lymphopoietin. No microbiota, cytokine, or inflammatory-biomarker measurements were available in this study.

Figure 1 provides a simplified biological framework. In allergic asthma, epithelial and immune signalling can contribute to airway inflammation and hyperresponsiveness; a human anti-TSLP antibody trial supports the relevance of this epithelial cytokine pathway [3]. Separately, experimental work in mice links dietary fibre, microbial short-chain fatty acids and altered allergic airway responses [4]. These findings provide biological context, but neither inflammatory activity nor microbiota was measured in the present survey.

Most early studies focused on individual nutrients. However, foods are consumed in combination with one another and may have synergetic effects; in fact, interest has shifted to the analysis of overall dietary patterns. Dietary patterns characterised by higher consumption of fruits, vegetables, legumes, and fish have been associated with more positive respiratory outcomes, while evidence regarding dietary patterns rich in processed foods and animal products has been less consistent [5,6].

The available data remain inconsistent, particularly among adults. Differences in study populations, dietary assessment methods, and the asthma outcomes considered have contributed to inconsistent results across studies [7,8]. In addition, few studies have investigated dietary patterns based on their potential inflammatory profile in large, nationally representative populations, and data on possible differences by sex and age are limited.

This study therefore examined the cross-sectional associations of two exploratory scores summarising selected food frequencies with self-reported asthma in adults participating in the 2023 Italian Aspects of Daily Life (AVQ) survey. The scores were not intended to measure systemic inflammation or to establish the inflammatory effects of individual foods.

## 2. Methods

### 2.1. Study Design and Data Source

We conducted a secondary cross-sectional analysis of public-use microdata from the 2023 Italian survey Aspetti della Vita Quotidiana (AVQ). AVQ is a nationwide annual household survey conducted by the Italian National Institute of Statistics to collect self-reported information on health, lifestyle, and socioeconomic conditions commonly used for research and epidemiological studies [9,10]. Survey expansion weights supplied in the public-use database were applied in all weighted analyses. The analysis used anonymised individual-level records, with no participant contact or biological sampling.

### 2.2. Study Population

The original file contained 41,750 records. After restriction to participants aged 18 years or older, 35,681 adults remained. Information on asthma was available for 33,182 participants; the putative pro-inflammatory and anti-inflammatory/protective scores were available for 35,133 and 35,242 participants, respectively. The complete-case sample used in multivariable regression analyses included 32,000 participants with complete information on asthma, both dietary scores, covariates, and survey weight. Thus, 3681 adults (10.3%) were excluded from regression analyses.

No a priori sample size calculation was performed for this secondary analysis; the survey sample size was determined by ISTAT independently of this research question. Precision is described by the reported 95% confidence intervals. The prevalence estimate refers to 33,182 adults with asthma information, whereas regression estimates refer to the 32,000-person complete-case sample; precision for subgroup analyses is not implied by the overall sample size.

### 2.3. Outcome Definition

The outcome was questionnaire-reported bronchial asthma, derived from the AVQ variable ASMA. In Section 7.4 of the questionnaire, respondents indicated whether they had each listed chronic condition, including bronchial asthma [11]. ASMA source code 4 was recoded as 1 and source code 3 as 0; missing responses remained missing. Here, “self-reported” means reported in the survey rather than verified through medical records, spirometry, or a clinician assessment. The item did not require confirmation of a physician diagnosis and did not distinguish asthma severity, phenotype, control, treatment, or age at onset. A separate questionnaire indicator identified chronic bronchitis/emphysema/respiratory insufficiency (BRON; source code 2 = present, 1 = absent). The corresponding sensitivity sample was restricted to participants explicitly reporting absence; those with missing BRON were also excluded. Family history of asthma, inflammatory biomarkers, and microbiota measurements was not available for this analysis.

### 2.4. Construction of Dietary Scores

Two exploratory, investigator-defined scores were constructed from selected AVQ food-frequency items. The PRO score comprised processed meats (SALUMI), beef (CBOV), pork excluding processed meats (CMAIAL), milk (LATTE), cheese and dairy products (FORM), eggs (UOVA), salty snacks (SNACK), and sweets (DOLCI). The ANTI score comprised fish (PESCE), leafy vegetables (VERD), the mixed item covering tomatoes and other vegetables (POMOD), fruit (FRUTTA), and dried or canned legumes (LEGUMI). POMOD does not measure tomatoes alone. The original labels “putative pro-inflammatory” (PRO) and “putative anti-inflammatory/protective” (ANTI) are retained only as shorthand for these exploratory food groupings, not as established biological properties.

**Figure 2:**
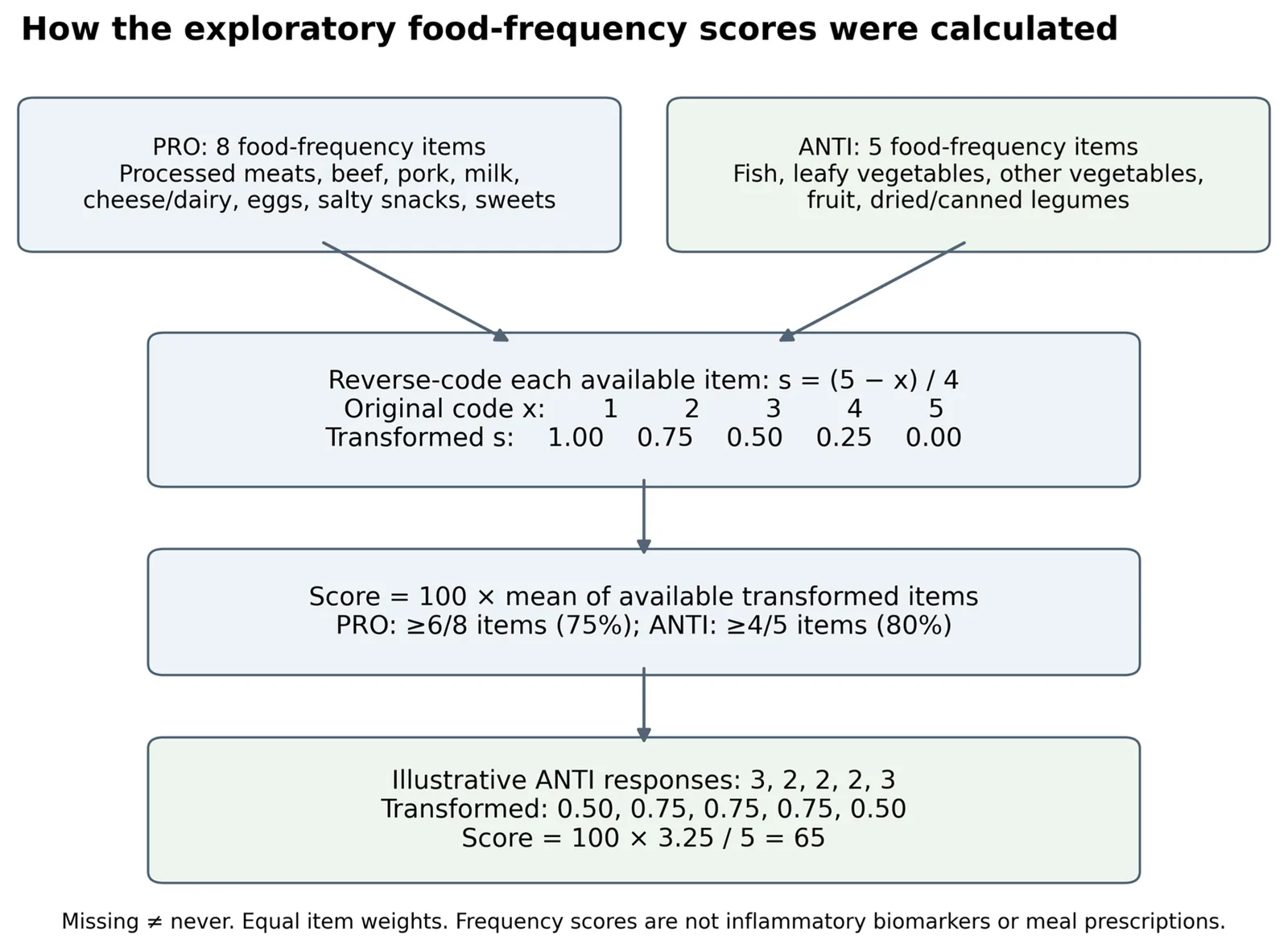
Construction of the exploratory PRO and ANTI scores. The ANTI example is hypothetical and illustrates arithmetic only. All selected items receive equal weights within a score. Scores are not dietary prescriptions, nutrient intakes, or calibrated inflammatory measures. Supplementary Tables S1–S3 give further details.

Food selection was guided by the available frequency items and the broad nutritional hypothesis contrasting selected animal-source/snack foods with plant foods and fish [5,6,7,8]. It was not a comprehensive assessment of diet quality. In particular, assigning milk, cheese, and eggs to PRO does not establish that these foods are intrinsically pro-inflammatory; the coarse categories cannot resolve food subtype, composition, preparation, or dietary context. Equal weights provide a transparent summary when defensible item-specific inflammatory coefficients are unavailable, but do not imply equal biological effects. The scores were not derived by factor analysis or calibrated against biomarkers and are not equivalent to the Dietary Inflammatory Index [12]. Figure 2 and Supplementary Tables S1–S3 provide the item inventory, scoring rules, and worked examples.

For each selected item, AVQ codes 1–5 were reverse-coded as s = (5 − x)/4, giving 1.00, 0.75, 0.50, 0.25, and 0.00, respectively. The ordered response categories run from more than once daily to never [11]. Adjacent ordinal categories were treated as equidistant; transformed values are not servings or actual daily frequencies. For score g in person i, Score_ig = 100 × Σ s_ij/m_ig, summing over the m_ig observed items. Missing items were omitted from the numerator and denominator, not coded as never. At least 6/8 PRO items or 4/5 ANTI items were required. These are the integer thresholds obtained by rounding up 70% of each item set (actual minimum coverage 75% and 80%); the rule is pragmatic, not a validated psychometric threshold. Higher scores indicate more frequent consumption of the corresponding selected foods. Both scores were also categorised using survey-weighted tertiles calculated among adults with the respective score available: PRO T1 ≤ 34.375, T2 > 34.375 to ≤43.750, and T3 > 43.750; ANTI T1 ≤ 50, T2 > 50 to ≤60, and T3 > 60. Ties at each cut point were assigned to the lower category, so category sizes need not be equal. Complete-case regression eligibility required calculable scores and complete covariates, not necessarily all 13 dietary responses. A reviewer-requested sensitivity analysis required valid responses to all 13 dietary items (8/8 PRO and 5/5 ANTI), retaining the original item coding, covariates, weights, and tertile boundaries.

### 2.5. Covariates

Covariates were selected a priori on the basis of the previous literature, epidemiological relevance, and their potential role as confounders [13,14,15]. The primary models included age group (18–44, 45–64, or ≥65 years), sex (male or female), education (primary/none, middle school, diploma, or university/postgraduate), BMI category (underweight, normal weight, overweight, or obese), smoking status (never, former, or current smoker), and self-reported household economic resources (excellent, adequate, scarce, or absolutely insufficient).

### 2.6. Statistical Analysis

Descriptive analyses summarised the sample, the dietary scores, and the weighted prevalence of self-reported asthma. Bivariate analyses compared participant characteristics according to asthma status using survey-weighted chi-square tests for categorical variables and survey-weighted *t*-tests for continuous dietary scores. The distribution of each dietary score across sociodemographic and lifestyle characteristics was examined using survey-weighted linear models.

Associations with asthma were estimated using survey-weighted logistic regression with a quasibinomial family. Separate adjusted models were fitted for the tertile-based and continuous parameterizations of each score. The categorical and continuous forms of the same score were never entered simultaneously in the same model. An additional mutually adjusted model included both dietary scores as continuous variables, expressed per 10-point increase, to estimate the association of each score independently of the other. All models were adjusted for age group, sex, education, BMI category, smoking status, and household economic resources. Adjusted odds ratios (ORs) and 95% confidence intervals (CIs) were reported.

The supplied expansion weight was COEFIN/10,000, implemented using svydesign (ids = ~1, weights = ~weight). Regression CIs were calculated as exp(β ± 1.96 × SE), matching the original code, and coefficient *p*-values used the survey residual degrees of freedom.

**Figure 3:**
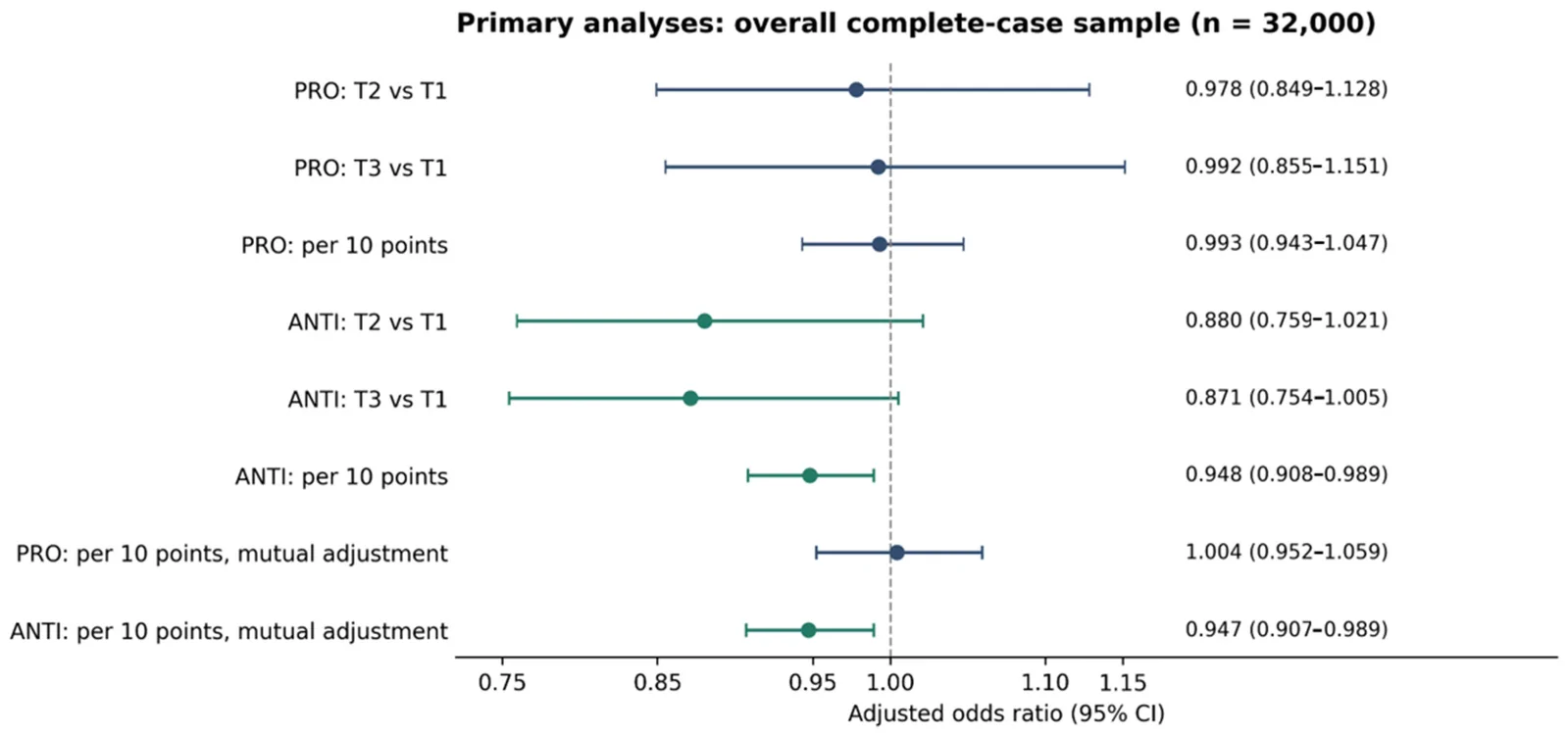
Primary overall-sample associations between exploratory dietary scores and self-reported asthma. Points and bars show adjusted ORs and 95% CIs from Table 1. Tertile and continuous specifications were fitted separately. The final two estimates are from the same mutually adjusted model. All continuous estimates are per 10 points. Subgroup and sensitivity results are reported separately in Table 5.

Primary analyses were the overall-sample models using separate tertile and continuous score specifications and the mutually adjusted continuous-score model (Table 1, Figure 3). Sex interactions and sex-stratified estimates were exploratory. Each interaction model included one score, sex, their product term, and the adjustment covariates; it did not include the other score. Sex-stratified models included both continuous scores. Sensitivity analyses repeated the mutually adjusted model in the following four separate restricted samples: excluding BRON+ participants; including only never smokers; including only adults aged 18–64 years; and including only adults aged 18–44 years. The age restrictions were intended to examine possible influence of older adults and respiratory diagnostic overlap, not to compare mutually exclusive age strata. The complete-item sensitivity repeated all five primary model specifications in participants with all 13 dietary responses (Table S4). All reported models used the three age groups where applicable. The original analyses used R version 4.2.3 (Copyright (C) 2023 The R Foundation for Statistical Computing) and the survey package 4.4.2. Revision-stage numerical replication and complete-item analyses used the same weighted estimating equations and sandwich variance in Python 3.12.14 (NumPy 2.3.5/SciPy 1.17.0), verified against the supplied R outputs; a corresponding R script is provided with the analysis materials. Two-sided *p*-values and 95% CIs are reported, with 0.05 as a conventional significance threshold. No multiplicity correction was applied; subgroup and sensitivity findings are hypothesis-generating. Continuous-score models assume a linear association with the log odds; non-linearity was not established by the reported analyses.

This observational cross-sectional study was reported in accordance with the Strengthening the Reporting of Observational Studies in Epidemiology (STROBE) statement [15]; the completed STROBE checklist is provided as Supplementary Material (Supplementary File S1).

## 3. Results

Among the 33,182 adults with non-missing asthma information, 1517 reported asthma, corresponding to a weighted prevalence of 4.62% (95% CI 4.37–4.89). The complete-case regression sample included 32,000 participants and 1460 asthma cases. Of the 35,681 adults, 3681 (10.3%) were excluded from regression analyses. Excluded participants differed from included participants with respect to age, education, BMI, smoking status, and household economic resources, but not sex. Among participants with non-missing asthma information, weighted asthma prevalence was similar in included and excluded participants (4.61% versus 4.91%; *p* = 0.687).

Participant characteristics according to asthma status are shown in Table 2. Asthma status was associated with sex (*p* = 0.005), age group (*p* < 0.001), education (*p* < 0.001), BMI category (*p* < 0.001), smoking status (*p* = 0.006), and household economic resources (*p* < 0.001). The weighted mean PRO score was 39.34 among participants with asthma and 39.71 among those without asthma (*p* = 0.319); the corresponding ANTI scores were 54.82 and 55.45 (*p* = 0.157). Weighted asthma prevalence did not differ significantly across tertiles of either the PRO score (*p* = 0.619) or the ANTI score (*p* = 0.503). Both dietary scores varied significantly across sociodemographic and lifestyle characteristics (Table 3). The PRO score was higher among men than women, decreased across increasing age groups, and was highest among current smokers. Conversely, the ANTI score was higher among women, increased with age, and was lower among current smokers. Global differences in the PRO score were statistically significant across sex, age group, education, BMI category, smoking status, and household economic resources. The ANTI score also differed significantly across all these characteristics.

**Table 1:** Main survey-weighted logistic regression models for self-reported asthma.

Model	Term	Adjusted OR	95% CI	*p*-Value
PRO tertiles	T2 vs. T1	0.978	0.849–1.128	0.763
PRO tertiles	T3 vs. T1	0.992	0.855–1.151	0.917
PRO continuous	Per 10-point increase	0.993	0.943–1.047	0.803
ANTI tertiles	T2 vs. T1	0.880	0.759–1.021	0.092
ANTI tertiles	T3 vs. T1	0.871	0.754–1.005	0.059
ANTI continuous	Per 10-point increase	0.948	0.908–0.989	0.014
PRO + ANTI continuous	PRO per 10-point increase	1.004	0.952–1.059	0.885
PRO + ANTI continuous	ANTI per 10-point increase	0.947	0.907–0.989	0.014

Models were adjusted for age group (18–44, 45–64, ≥65 years), sex, education, BMI category, smoking, and household economic resources. Separate models were fitted for PRO tertiles, PRO continuous, ANTI tertiles, ANTI continuous, and mutually adjusted PRO + ANTI continuous. The two contrasts within a tertile model share the same model; the two mutually adjusted estimates also share one model. Continuous and categorical forms of the same score were never entered together. T1 is the reference category.

**Table 2:** Characteristics of the complete-case study population according to self-reported asthma status.

Characteristic	Category	No Asthma (n = 30,540), n (Weighted %)	Asthma (n = 1460), n (Weighted %)	*p*-Value
Sex	Female	16,056 (51.6)	817 (55.9)	0.005
	Male	14,484 (48.4)	643 (44.1)	
Age group	18–44	10,368 (36.9)	344 (26.8)	<0.001
	45–64	11,133 (36.0)	487 (33.7)	
	65+	9039 (27.0)	629 (39.6)	
Education	Diploma	12,609 (41.5)	485 (33.6)	<0.001
	Middle school	8226 (27.1)	428 (29.5)	
	Primary/none	4095 (12.4)	351 (22.1)	
	University/postgrad	5610 (19.0)	196 (14.8)	
BMI category	Normal	15,678 (51.2)	620 (43.6)	<0.001
	Obese	3447 (11.2)	242 (17.0)	
	Overweight	10,325 (34.0)	557 (37.2)	
	Underweight	1090 (3.6)	41 (2.3)	
Smoking status	Current	5850 (20.2)	249 (17.5)	0.006
	Former	7752 (25.3)	438 (29.0)	
	Never	16,938 (54.5)	773 (53.5)	
Household economic resources	Absolutely insufficient	913 (3.3)	66 (4.6)	<0.001
	Adequate	20,503 (66.3)	878 (60.1)	
	Excellent	533 (1.9)	23 (1.3)	
	Scarce	8591 (28.5)	493 (34.0)	
PRO score tertile	T1	10,908 (36.0)	552 (37.4)	0.619
	T2	9998 (32.8)	468 (32.5)	
	T3	9634 (31.1)	440 (30.1)	
ANTI score tertile	T1	12,155 (40.3)	609 (42.0)	0.503
	T2	8696 (28.3)	411 (27.6)	
	T3	9689 (31.4)	440 (30.4)	
PRO score, mean (SD)		39.71 (11.84)	39.34 (12.05)	0.319
ANTI score, mean (SD)		55.45 (14.36)	54.82 (14.69)	0.157

Values are unweighted counts with survey-weighted column percentages unless otherwise indicated. Continuous scores are survey-weighted mean (SD). *p*-values are from survey-weighted chi-square tests or survey-weighted *t*-tests, as appropriate.

**Table 3:** Distribution of dietary scores according to participant characteristics.

Characteristic	Category	PRO Score, Mean (SD)	ANTI Score, Mean (SD)	*p* PRO	*p* ANTI
Sex	Female	39.11 (11.81)	56.83 (14.05)	<0.001	<0.001
	Male	40.32 (11.87)	53.90 (14.57)		
Age group	18–44	41.57 (12.05)	53.42 (15.33)	<0.001	<0.001
	45–64	39.11 (11.75)	55.51 (14.15)		
	65+	37.96 (11.37)	57.95 (12.89)		
Education	Diploma	40.06 (11.74)	54.64 (14.73)	<0.001	<0.001
	Middle school	40.53 (12.12)	55.13 (14.71)		
	Primary/none	38.82 (11.81)	56.50 (13.62)		
	University/postgrad	38.27 (11.56)	56.79 (13.42)		
BMI category	Normal	39.36 (11.87)	55.41 (14.51)	<0.001	0.003
	Obese	40.43 (12.12)	55.09 (14.77)		
	Overweight	40.04 (11.53)	55.70 (13.91)		
	Underweight	38.77 (13.44)	53.83 (15.50)		
Smoking status	Current	40.54 (12.07)	52.31 (15.01)	<0.001	<0.001
	Former	39.02 (11.65)	56.22 (13.70)		
	Never	39.70 (11.85)	56.19 (14.29)		
Household economic resources	Absolutely insufficient	38.25 (13.65)	52.39 (17.05)	0.016	<0.001
	Adequate	39.74 (11.63)	55.92 (13.94)		
	Excellent	40.55 (13.82)	56.89 (15.73)		
	Scarce	39.69 (11.98)	54.52 (14.83)		

Values are survey-weighted means (SDs). *p*-values are global tests from survey-weighted linear models comparing each score across categories of one characteristic at a time; they are not pairwise comparisons. PRO and ANTI are exploratory food-frequency scores.

### 3.1. Main Regression Models

In adjusted overall-sample models, PRO was not associated with self-reported asthma in either parameterisation (Table 1, Figure 3). For ANTI, the OR was 0.880 (95% CI 0.759–1.021; *p* = 0.092) for T2 versus T1 and 0.871 (95% CI 0.754–1.005; *p* = 0.059) for T3 versus T1. In the separate continuous model, the OR per 10 points was 0.948 (95% CI 0.908–0.989; *p* = 0.014).

In the mutually adjusted continuous-score model, PRO had an OR of 1.004 (95% CI 0.952–1.059; *p* = 0.885), and ANTI an OR of 0.947 (95% CI 0.907–0.989; *p* = 0.014). The latter corresponds to approximately 5.3% lower adjusted odds per 10 points, not a 5.3 percentage-point reduction in prevalence or an estimate of causal risk reduction. The complete model is reported in Table 4.

### 3.2. Exploratory Interaction, Stratified, and Sensitivity Analyses

Exploratory sex-interaction estimates were 0.875 for PRO × female sex (95% CI 0.790–0.970; *p* = 0.011) and 1.004 for ANTI × female sex (95% CI 0.922–1.093; *p* = 0.925). These are ratios of score-associated ORs, not direct associations in women. In separate sex-stratified models, male estimates were 1.096 (95% CI 1.015–1.183; *p* = 0.020) for PRO and 0.925 (95% CI 0.866–0.988; *p* = 0.021) for ANTI; female estimates were 0.937 (95% CI 0.870–1.009; *p* = 0.084) and 0.962 (95% CI 0.908–1.019; *p* = 0.183), respectively. A significant association in one sex and a non-significant association in the other does not itself establish interaction; in particular, the ANTI interaction provided no evidence of a sex difference (Table 5).

After exclusion of BRON+ participants, the ANTI estimate was farther below 1 (OR 0.912, 95% CI 0.862–0.965; *p* = 0.001). Restriction to ages 18–64 years yielded OR 0.946 (95% CI 0.895–0.999; *p* = 0.046), while the CIs included one in never smokers and in adults aged 18–44 years. PRO estimates were non-significant in each restricted sample. These analyses were secondary and do not demonstrate differences between populations (Table 5).

Requiring all 13 dietary items retained 30,474 participants and 1369 asthma cases, excluding 1526 participants (4.8%) and 91 cases from the primary regression sample. In the mutually adjusted model, the OR per 10 points was 1.006 (95% CI 0.952–1.064; *p* = 0.831) for PRO and 0.952 (95% CI 0.910–0.995; *p* = 0.031) for ANTI. Separate continuous models gave similar estimates; ANTI tertile contrasts remained non-significant (T2 vs. T1: OR 0.893, 95% CI 0.766–1.040; *p* = 0.145; T3 vs. T1: OR 0.881, 95% CI 0.759–1.023; *p* = 0.097). Complete results are presented in Table S4.

**Table 5:** Exploratory sex-interaction models and mutually adjusted sex-stratified and restricted-sample analyses.

Analysis/Stratum	Term	Adjusted OR	95% CI	*p*-Value
Sex interaction	PRO × female sex	0.875	0.790–0.970	0.011
Sex interaction	ANTI × female sex	1.004	0.922–1.093	0.925
Male	PRO per 10 points	1.096	1.015–1.183	0.020
Male	ANTI per 10 points	0.925	0.866–0.988	0.021
Female	PRO per 10 points	0.937	0.870–1.009	0.084
Female	ANTI per 10 points	0.962	0.908–1.019	0.183
Sensitivity: excluding BRON+	PRO per 10 points	1.028	0.956–1.105	0.454
Sensitivity: excluding BRON+	ANTI per 10 points	0.912	0.862–0.965	0.001
Sensitivity: never smokers only	PRO per 10 points	0.992	0.924–1.065	0.831
Sensitivity: never smokers only	ANTI per 10 points	0.970	0.913–1.031	0.327
Restricted to ages 18–64 years	PRO per 10 points	1.024	0.957–1.096	0.489
Restricted to ages 18–64 years	ANTI per 10 points	0.946	0.895–0.999	0.046
Restricted to ages 18–44 years	PRO per 10 points	1.033	0.934–1.143	0.524
Restricted to ages 18–44 years	ANTI per 10 points	0.952	0.878–1.031	0.226

BRON+: self-reported chronic bronchitis, emphysema, or respiratory insufficiency. All continuous estimates are per 10 points. Interaction models included one score at a time and its interaction with sex, with adjustment for the remaining covariates. Sex-stratified and restricted-sample models included both scores and the applicable covariates. Invariant covariates were omitted within strata. The BRON sensitivity required an explicit negative BRON response. The complete-dietary-item sensitivity is reported separately in Table S4. Interaction terms are ratios of ORs on the multiplicative scale and are distinct from the separate stratum-specific model estimates. The four sensitivity samples overlap and are not independent replications. *p*-values are unadjusted for multiplicity. Age restrictions are not mutually exclusive age-stratified comparisons.

## 4. Discussion

In this secondary cross-sectional analysis, the continuous ANTI score was modestly inversely associated with self-reported asthma after covariate adjustment, with little change after mutual adjustment for PRO. PRO showed no overall association. These results concern selected food-frequency patterns, not measured systemic inflammation, clinically confirmed asthma, or the effect of changing diet.

Evidence for ANTI depended on parameterisation. The tertile estimates were below 1 and therefore directionally compatible with the continuous estimate, but both CIs included 1. Categorisation discards within-category information and may reduce precision, whereas the continuous model imposes a linear log-odds relationship. The difference in *p*-values is not proof of a linear dose–response, non-linearity, or a dietary threshold. Given the small association, the non-significant tertile contrasts, and the lack of significant crude score differences, the finding should be regarded as limited evidence requiring replication rather than a consistently protective pattern. Adjustment may address differences in participant profiles but cannot eliminate residual confounding.

Observational studies of overall dietary patterns and asthma provide a relevant comparison [7,8,13,14,15], but their instruments and outcomes differ from those used here. Our score is not interchangeable with validated diet-quality or inflammatory indices. The gut–lung axis provides a possible biological hypothesis: experimental fibre interventions in mice altered microbial metabolites and allergic airway responses [4]. However, AVQ provides no paired microbiota profiles or inflammatory biomarkers for this analysis. We therefore could not estimate diet–microbiota or inflammation–asthma correlations, investigate mediation, or test whether inflammation explains the observed association. The literature-based schematic in Figure 1 should not be interpreted as a mechanism demonstrated by our data.

Sex-specific findings are exploratory. Multiple interaction, subgroup, and sensitivity comparisons were examined without multiplicity adjustment, increasing the possibility of chance findings. The PRO interaction warrants replication but does not establish a sex-specific causal effect. For ANTI, the non-significant interaction does not support a sex difference, despite a nominally significant association among men alone. Differences in diet, residual confounding, and imprecision may contribute to the subgroup estimates; hormonal or menopause-related mechanisms cannot be inferred from these data.

The restricted-sample estimates offer only partial reassurance. Exclusion of BRON+ participants may reduce some diagnostic overlap but also changes the population and cannot validate asthma diagnoses or establish the source of the larger inverse estimate. The ANTI estimate among adults aged 18–64 years was similar to the main estimate; the 18–44-year estimate also had a similar direction and magnitude but a wider CI that included 1. Differences in statistical significance should not be interpreted as evidence that an association is absent in younger adults, or that the association differs by age. These overlapping analyses do not provide independent replications.

Several limitations are central to interpretation. First, the cross-sectional design cannot establish temporal order or causation: people with asthma may change diet after diagnosis or symptoms. Second, questionnaire-reported asthma can include false-positive and false-negative classifications, including overlap with other respiratory conditions. No clinical validation, asthma-specific medication history, severity, phenotype, or control data were available. The direction and magnitude of any resulting bias cannot be determined. Family history was unavailable for adjustment, leaving possible residual confounding by familial susceptibility and shared behaviours. Third, the scores are unvalidated summaries of selected ordinal food frequencies. Equal weighting, equidistant coding, food grouping, omitted dietary dimensions, and the absence of item-specific quantities and total energy intake limit nutritional interpretation. In particular, broad dairy, egg, and snack items cannot be assumed to share a uniform inflammatory effect. Neither score is a validated overall diet-quality measure. Fourth, available-item averaging can make scores depend on which foods are missing; the 6/8 and 4/5 thresholds do not eliminate this problem. The complete-item analysis yielded a similar continuous ANTI estimate, providing some reassurance about the availability rule within the analysed sample. However, it does not establish that dietary missingness is ignorable, validate the scores, or remove selection bias. Fifth, exclusion of 10.3% of adults and differences between included and excluded participants may introduce selection bias; similar asthma prevalence in the observed subset does not rule this out. Finally, residual confounding by factors not included in the models, including physical activity, environmental and occupational exposures, and healthcare access, remains possible. Survey weights do not remove these limitations or the possibility of dietary reporting error.

Strengths include the large national survey sample, application of survey weights, transparent reporting of all five main model specifications, and presentation of both statistically significant and non-significant secondary estimates. These features support descriptive population relevance but do not validate the scores or establish biological or clinical effects.

Generalisability is primarily to the household population represented by AVQ, subject to survey participation and complete-case selection. National sampling and weighting do not automatically establish applicability to other countries or to clinical asthma populations. Replication with comparable and independently validated measurements is needed.

## 5. Conclusions

In conclusion, a higher continuous ANTI food-frequency score was associated with modestly lower adjusted odds of self-reported asthma, whereas tertile contrasts were non-significant and PRO showed no overall association. These exploratory cross-sectional findings do not establish an inflammatory mechanism, reduced asthma risk, or respiratory benefit. Sex-specific findings require independent replication. Longitudinal studies using validated dietary measures and clinically characterised asthma are needed.

## Figures and Tables

**Table 4:** Full mutually adjusted survey-weighted logistic regression model for self-reported asthma.

Variable	Comparison	Adjusted OR	95% CI	*p*-Value
PRO score	Per 10-point increase	1.004	0.952–1.059	0.885
ANTI score	Per 10-point increase	0.947	0.907–0.989	0.014
Age group	45–64 vs. 18–44	1.164	0.987–1.372	0.071
Age group	65+ vs. 18–44	1.542	1.289–1.844	<0.001
Sex	Female vs. Male	1.218	1.071–1.384	0.003
Education	Primary/none vs. University/postgrad	1.521	1.215–1.904	<0.001
Education	Middle school vs. University/postgrad	1.175	0.957–1.442	0.123
Education	Diploma vs. University/postgrad	0.968	0.796–1.178	0.748
BMI category	Underweight vs. Normal	0.733	0.502–1.070	0.107
BMI category	Overweight vs. Normal	1.184	1.031–1.359	0.017
BMI category	Obese vs. Normal	1.571	1.312–1.881	<0.001
Smoking status	Former vs. Never	1.147	0.995–1.322	0.058
Smoking status	Current vs. Never	0.974	0.820–1.158	0.768
Household economic resources	Excellent vs. Adequate	0.799	0.487–1.312	0.375
Household economic resources	Scarce vs. Adequate	1.259	1.105–1.434	<0.001
Household economic resources	Absolutely insufficient vs. Adequate	1.465	1.080–1.988	0.014

## Data Availability

The source public-use AVQ microdata are available from the Italian National Institute of Statistics (ISTAT), https://www.istat.it/en/microdata/aspects-of-daily-life/ (accessed on 29 June 2026). The present study analysed existing data; it did not collect new participant-level data. The score definitions and illustrative calculations are provided in the Supplementary Materials.

## Supplementary Materials

The following supporting information can be downloaded at https://www.mdpi.com/article/10.3390/nu18213485/s1, Supplementary File S1 contains the updated STROBE checklist; Table S1: Items included in each score; Table S2: Response transformation and availability rules; Table S3: Hypothetical calculations (not participant data); Table S4: Sensitivity analysis requiring all 13 dietary items.

## Abbreviations

ANTI, exploratory putative anti-inflammatory/protective food-frequency score; AVQ, Aspetti della Vita Quotidiana; BMI, body mass index; BRON+, chronic bronchitis/emphysema/respiratory insufficiency; CI, confidence interval; COPD, chronic obstructive pulmonary disease; ISTAT, Italian National Institute of Statistics; OR, odds ratio; PRO, exploratory putative pro-inflammatory food-frequency score; SD, standard deviation; TSLP, thymic stromal lymphopoietin.
