# Supplementary material for: Exploratory Food-Frequency Scores and Self-Reported Asthma in Italian Adults: A Nationwide Cross-Sectional Study
Source: Nutrients. 2026 Oct 23;18(21):3485. doi: 10.3390/nu18213485 (PMC13765357; doi:10.3390/nu18213485)
Supplement: Supplementary file 1 [file nutrients-18-03485-s001.zip › nutrients-4610568-supplementary.pdf]

Supplementary Material

Exploratory Food-Frequency Scores and Self-Reported Asthma in Italian Adults: A Nationwide Cross-Sectional Study

STROBE checklist for cross-sectional studies

Locations refer to section headings and tables rather than unstable page numbers. Outstanding reporting limitations are stated explicitly; this checklist is not a claim that every recommended analysis was performed.

Section/topic	Item	Recommendation	Location / qualification
Title and abstract	1	(a) Indicate the study's design with a commonly used term in the title or the abstract	Title and structured Abstract: cross-sectional design; exploratory scores; cautious conclusions.
		(b) Provide an informative and balanced summary of what was done and what was found	Structured Abstract; see item 1 above.
Introduction — Background/rationale	2	Explain the scientific background and rationale for the investigation being reported	Introduction and Figure 1: background and limits of biological inference.
Introduction — Objectives	3	State specific objectives, including any prespecified hypotheses	Introduction, final paragraph: objective. The analysis is exploratory; no new preregistration is claimed.
Methods — Study design	4	Present key elements of study design early in the paper	Sections 2.1–2.2: secondary cross-sectional analysis.
Methods — Setting	5	Describe the setting, locations, and relevant dates, including periods of recruitment, exposure, follow-up, and data collection	Section 2.1: Italian AVQ 2023 survey; source questionnaire cited.
Methods — Participants	6	(a) Give eligibility criteria, and the sources and methods of selection of participants	Section 2.2: age restriction, score availability, and complete-case eligibility.
Methods — Variables	7	Clearly define all outcomes, exposures, predictors, potential confounders, and effect modifiers	Sections 2.3–2.5; Tables S1–S3: outcome, exposures, and adjustment variables.
Methods — Data sources/measurement	8	For each variable of interest, give sources of data and details of methods of assessment	Sections 2.3–2.4; questionnaire reference; Tables S1–S3.
Methods — Bias	9	Describe any efforts to address potential sources of bias	Sections 2.4 and 2.6; Discussion: misclassification, missingness, residual confounding and selection.
Methods — Study size	10	Explain how the study size was arrived at	Section 2.2: existing national survey; no study-specific a priori sample-size calculation.
Methods — Quantitative variables	11	Explain how quantitative variables were handled in the analyses; describe grouping and why	Sections 2.4–2.6: ordinal transformation, continuous scores, exact weighted tertile cut points and handling of ties, and age grouping.
Methods — Statistical methods	12	(a)-(e) Describe all statistical methods, subgroups/interactions, missing data handling, sampling strategy, sensitivity analyses	Section 2.6: weighted models/tests, interactions, restricted samples and complete-item sensitivity (Table S4).
Results — Participants	13	(a)-(c) Numbers at each stage, reasons for non-participation, flow diagram	Section 2.2 and Results: 41,750 records, 35,681 adults, 33,182 with asthma information, 32,000 primary regression participants; 30,474 with all 13 items. Missingness-related exclusions may overlap.

Section/topic	Item	Recommendation	Location / qualification
Results — Descriptive data	14	(a)-(c) Characteristics of participants, exposure/confounder data, missing data	Tables 1–2 and Results: participant characteristics and scores. Aggregate missingness/selection described; item-level missingness distribution is not reported.
Results — Outcome data	15	Report numbers of outcome events	Results opening paragraph: 1,517 asthma reports overall and 1,460 in the regression sample.
Results — Main results	16	(a)-(c) Unadjusted and adjusted estimates and precision; category boundaries; translate relative risk to absolute risk if relevant	Table 3/Figure 3 and Table 4: adjusted ORs/CIs; Table 1: crude descriptive comparisons. Section 2.4 gives exact tertile boundaries. Unadjusted logistic ORs are not reported; no causal absolute-risk translation is attempted.
Results — Other analyses	17	Report other analyses done (subgroups, interactions, sensitivity)	Section 3.2, Table 5 and Table S4: sex analyses, four original restricted samples and additional complete-item sensitivity.
Discussion — Key results	18	Summarize key results with reference to study objectives	Discussion opening paragraph: principal findings.
Discussion — Limitations	19	Discuss limitations, sources of bias/imprecision, direction and magnitude of bias	Discussion: unvalidated scores, self-report, reverse causation, missingness, selection, confounding and imprecision; direction/magnitude of bias cannot be quantified.
Discussion — Interpretation	20	Cautious overall interpretation considering objectives, limitations, multiplicity, similar studies	Discussion and Conclusion: cautious interpretation of parameterisation and multiplicity.
Discussion — Generalisability	21	Discuss generalisability of the study results	Discussion, generalisability paragraph: household survey population and complete-case limits.
Other information — Funding	22	Give the source of funding and the role of funders	Funding statement: no specific funding.

Dietary score definitions and examples

PRO and ANTI retain the original exploratory labels for traceability. They measure frequency summaries, not calibrated inflammatory potential or a validated healthy-diet construct. The grouping and all reported effect estimates have been retained; no revised scoring model has been fitted.

Table S1. Items included in each score

Score	AVQ variable	Food-frequency item
PRO	SALUMI	Processed meats/salami products
PRO	CBOV	Beef
PRO	CMAIAL	Pork, excluding processed meats
PRO	LATTE	Milk
PRO	FORM	Cheese and dairy products
PRO	UOVA	Eggs
PRO	SNACK	Salty snacks
PRO	DOLCI	Sweets
ANTI	PESCE	Fish
ANTI	VERD	Leafy vegetables
ANTI	POMOD	Tomatoes and other vegetables, not tomatoes alone
ANTI	FRUTTA	Fruit
ANTI	LEGUMI	Dried or canned legumes

Rationale and limitations: ANTI summarises selected plant-food and fish frequencies; PRO groups selected animal-source foods and snacks. This is a pragmatic grouping, not a validated classification of the biological effects of each food. Broad dairy, egg, and snack categories contain heterogeneous products; equal weights cannot account for those differences. The selected items do not exhaust the diet and do not quantify total energy or nutrient intake. POMOD is a mixed vegetable item; its name must not be interpreted as a tomato-specific exposure.

Table S2. Response transformation and availability rules

Original code	Consumption frequency	Transformed item score
1	More than once daily	1.00
2	Once daily	0.75
3	A few times per week	0.50
4	Less than once per week	0.25
5	Never	0.00
Missing	No observed response	Missing; excluded from item mean

Transformation: $s = (5 - x)/4$. Person-level score: 100 × sum of available s values / number of available items. PRO requires at least 6 of 8 items; ANTI requires at least 4 of 5. These thresholds correspond to $\text{ceil}(0.70 \times 8) = 6$ and $\text{ceil}(0.70 \times 5) = 4$, giving actual coverage of 75% and 80%. This common fractional rule does not provide psychometric validation. Scores based on incomplete item sets may not be directly comparable if missingness is informative.

Table S3. Hypothetical calculations (not participant data)

Example	Original codes in Table S1 order	Calculation	Result
PRO, all items	3, 3, 4, 2, 3, 4, 5, 3	100 × (0.50 + 0.50 + 0.25 + 0.75 + 0.50 + 0.25 + 0 + 0.50) / 8	40.625
ANTI, all items	3, 2, 2, 2, 3	100 × (0.50 + 0.75 + 0.75 + 0.75 + 0.50) / 5	65.00
ANTI, one item missing	3, 2, missing, 2, 3	100 × (0.50 + 0.75 + 0.75 + 0.50) / 4	62.50
PRO, only 5 items observed	3, 3, 4, 2, 3, missing, missing, missing	5 < 6 required items	Not calculated

The examples illustrate arithmetic and missing-value handling only. They are not observed participants, typical diets, meal plans, or dietary recommendations. The 0–100 score range does not imply a probability, percentage of inflammation, or clinical threshold. The regression complete-case sample can still contain participants with one or two missing dietary items when the availability rule is met. Results requiring all 13 valid dietary responses are reported in Table S4; the score definitions and original tertile cut points were retained.

Table S4. Sensitivity analysis requiring all 13 dietary items

The restricted sample included 30,474 adults and 1,369 self-reported asthma cases (95.2% of the primary regression sample). The additional restriction removed 1,526 adults and 91 cases. All five primary specifications were repeated using the original expansion weights, adjustment covariates, and tertile boundaries. Both scores were based on all their items; no missing dietary response was imputed.

Model	Term	Adjusted OR	95% CI	p-value
PRO tertiles	T2 vs T1	0.990	0.855–1.147	0.897
PRO tertiles	T3 vs T1	0.996	0.854–1.162	0.962
PRO continuous	PRO per 10 points	0.997	0.944–1.053	0.914
ANTI tertiles	T2 vs T1	0.893	0.766–1.040	0.145
ANTI tertiles	T3 vs T1	0.881	0.759–1.023	0.097
ANTI continuous	ANTI per 10 points	0.953	0.911–0.996	0.031
PRO + ANTI continuous	PRO per 10 points	1.006	0.952–1.064	0.831
PRO + ANTI continuous	ANTI per 10 points	0.952	0.910–0.995	0.031

Adjustment: age group (18–44, 45–64, ≥65 years), sex, education, BMI category, smoking status, and household economic resources. The two final rows belong to one mutually adjusted model. Tertile cut points were not re-estimated after restriction. CIs use 1.96 standard errors; p-values use residual degrees of freedom. These are secondary sensitivity analyses with no multiplicity adjustment. The primary comparator estimates are in main Table 3.
